# An Antifungal Role of Hydrogen Sulfide on the Postharvest Pathogens *Aspergillus niger* and *Penicillium italicum*

**DOI:** 10.1371/journal.pone.0104206

**Published:** 2014-08-07

**Authors:** Liu-Hui Fu, Kang-Di Hu, Lan-Ying Hu, Yan-Hong Li, Liang-Bin Hu, Hong Yan, Yong-Sheng Liu, Hua Zhang

**Affiliations:** 1 School of Biotechnology and Food Engineering, Hefei University of Technology, Hefei, China; 2 School of Food Science, Henan Institute of Science and Technology, Xinxiang, China; 3 College of Chemical and Environmental Engineering, Harbin University of Science and Technology, Key Laboratory of Green Chemical Technology of College of Heilongjiang Province, Harbin, China; Emory University, United States of America

## Abstract

In this research, the antifungal role of hydrogen sulfide (H_2_S) on the postharvest pathogens *Aspergillus niger* and *Penicillium italicum* growing on fruits and under culture conditions on defined media was investigated. Our results show that H_2_S, released by sodium hydrosulfide (NaHS) effectively reduced the postharvest decay of fruits induced by *A. niger* and *P. italicum*. Furthermore, H_2_S inhibited spore germination, germ tube elongation, mycelial growth, and produced abnormal mycelial contractions when the fungi were grown on defined media in Petri plates. Further studies showed that H_2_S could cause an increase in intracellular reactive oxygen species (ROS) in *A. niger*. In accordance with this observation we show that enzyme activities and the expression of superoxide dismutase (SOD) and catalase (CAT) genes in *A. niger* treated with H_2_S were lower than those in control. Moreover, H_2_S also significantly inhibited the growth of *Saccharomyces cerevisiae*, *Rhizopus oryzae*, the human pathogen *Candida albicans*, and several food-borne bacteria. We also found that short time exposure of H_2_S showed a microbicidal role rather than just inhibiting the growth of microbes. Taken together, this study suggests the potential value of H_2_S in reducing postharvest loss and food spoilage caused by microbe propagation.

## Introduction

Generally, about 20% of harvested fruits and vegetables undergo decay during postharvest storage [1]. Considerable postharvest decay is caused by plant fungal pathogens [2]. It has been reported that *Aspergillus niger* can induce the spoilage of fruits such as cherry tomatoes and grapes, and *Penicillium italicum* can cause postharvest blue mold of citrus fruit [3], [4], [5]. Decay caused by food-borne bacterial pathogens AR also a major concern due to the increasing demands for food safety [6]. For instance, both *Salmonella typhimurium* and *Staphylococcus aureus* in contaminated food are leading causes of gastroenteritis [7], [8]. The application of synthetic chemical as germicides is a primary method to control postharvest decay [9]. However, chemical control faces two intractable problems: first, the inevitable development of pathogen resistance; and second, a range of generally used germicides are under review in many countries due to health safety issues [10]. Thus, there is a growing need to develop alternative treatments of postharvest disease that are more enduring and safe.

Hydrogen sulfide (H_2_S), traditionally thought as a toxic gas, has proved to be a gaseous signaling molecule after nitric oxide and carbon monoxide in animals [11]. Accumulating evidence shows multiple roles of H_2_S in plant development, abiotic stresses, and postharvest senescence [12], [13], [14], [15], [16]. Nitric oxide has also been shown to extend postharvest storage of fruits and to inhibit the growth of postharvest pathogens [17], [18]. Lai et al. [19] found that the inhibitory effect of NO on the spores of *Penicillium expansum* was associated with oxidative damage. Similarly, it has been found that exogenous H_2_S application can prolong postharvest storage of strawberry, fresh-cut kiwifruit, broccoli and mulberry fruit by modulating the antioxidant system [15], [20], [21], [22]. The concentration of the applied H_2_S required to delay senescence in strawberry is quite low, indicating that fumigation of fruits with H_2_S gas could be safe and practical [15]. However, there is limited data on the relations between H_2_S and postharvest pathogens. The earliest related research on this topic was reported by Marsh [23] who found that H_2_S was toxic to germinating spores of *Sclerotinia fructicola*. Recently, Hu et al. [24] showed that H_2_S could prolong postharvest storage of fresh-cut pears and inhibit fungal growth, although the underlining mechanism of the antifungal role of H_2_S is unknown.

In this work, we investigated the antifungal effect of H_2_S on the postharvest pathogens *A. niger* and *P. italicum* inoculated on fruits, as well as on the growth of these fungi on Petri dishes with defined media. We also examined the effect of H_2_S on baker's yeast (*Saccharomyces cerevisiae*), *Rhizopus oryzae*, the human pathogen *Candida albicans*, and several food-borne bacteria, including *Staphyloccocus aureus*, *Salmonella typhimurium*, *Listeria monocytogenes*, *Bacillus subtilis*, *Bacillus thuringiensis*, *Escherichia coli* and *Enterobacter aerogenes*.

## Materials and methods

### Materials

Six different fruits, apple (*Malus domestica*), kiwifruit (*Actinidia deliciosa*), pear (*Pyrus bretschneideri* Rehd.), sweet orange (*Citrus sinensis*), mandarin (*Citrus reticulata*) and tomato (*Lycopersicon esculentum*), used in this work were supplied by a fruit market in Hefei, Anhui province, China. Unwounded and healthy fruits, all of a similar size and maturity, were selected for experimentation. Pure fungal and bacterial isolates used in this research were kindly supplied by School of Biotechnology and Food Engineering, Hefei University of Technology, Anhui, People's Republic of China, except *Candida albicans* (SC5314) which was kindly bestowed by Prof. Jianli Sang at College of Life Science, Beijing Normal University. Three molds (*Aspergillus niger*, *Penicillium italicum*, *Rhizopus oryzae*), two yeasts (*Saccharomyces cerevisiae*, *Candida albicans*) and seven bacteria (*Staphyloccocus aureus*, *Salmonella typhimurium*, *Listeria monocytogenes*, *Bacillus subtilis*, *Bacillus thuringiensis*, *Escherichia coli* and *Enterobacter aerogenes*) were used in this study. Molds, yeasts and bacteria were cultured on potato dextrose agar (PDA), yeast peptone dextrose (YPD) agar and nutrient agar in Petri dishes, respectively. Sodium hydrosulfide (NaHS) was purchased from Sigma and used as exogenous H_2_S donor [24]. Aqueous NaHS solutions (150 mL) at different concentrations were placed in the bottom of sealed containers (volume 3 L) to release H_2_S to fumigate fruits or pathogens. Aqueous NaHS solutions could steadily release H_2_S gas since 30 min, and almost keep at the same level till 24 h. Thus NaHS solutions are renewed daily.

### Antifungal Assay of H_2_S on Postharvest Fruits Infected with *A. niger* and *P. italicum*

*A. niger* and *P. italicum* spores were harvested from isolates after 6 d growth at 25°C, and suspended in sterile physiological saline. The suspensions were then filtered through sterile gauze to remove mycelium and adjusted to 1×10^6^ spores per ml. Apples, kiwifruits, pears, sweet oranges, mandarins and tomatoes were washed with tap water and sterilized with 75% ethanol. The surfaces of these fruits were wounded at five different sites and each of the wounds (2 mm diameter and 4 mm deep) was injected with 5 µL of spore suspension [25]. After air-drying, three replicates of plant samples were fumigated with 0.5 mM NaHS solution (150 mL) or H_2_O (as control) in sealed containers at 25°C. NaHS solutions were changed daily.

### Antifungal Effects of H_2_S on *A. niger* and *P. italicum*

A 2 µL spore suspension (1×10^6^ spores per mL) was inoculated in the centre of a 9-cm diameter Petri dish and NaHS solutions at different concentrations (0.00, 0.01, 0.05, 0.10, 0.50 and 2.5 mM) were added in the bottom of sealed containers to fumigate the pathogens at 25°C and the NaHS solutions were renewed daily. The colony diameters (mm) of *A. niger* and *P. italicum* were recorded for 8 d. The lowest NaHS concentration that resulted in failure of colony formation after 4 d incubation was regarded as the minimal inhibitory concentration (MIC) [26]. To investigate whether H_2_S treatment could kill the pathogens or just inhibit their growth, *A. niger* and *P. italicum* were first treated with 2.5 mM NaHS solution for 1 day and allowed to recover with water fumigation for another 8 d at 25°C.

### Determination of Spore Germination and Germ Tube Elongation

Spore germination and germ tube elongation was measured according to Lazar et al. [18] and Liu et al. [26] with minor modifications. 20 µL of spore suspension (1×10^6^ spores per ml) was placed on a 7-mm diameter plug of PDA which was then placed on a glass slide. Each plug-slide was held in a Petri dish with moistened filter paper, and then placed in sealed container at 25°C. Spore germination (germ tube longer than two times the diameter of the corresponding spore) was assessed under a microscope on three occasions (6, 12 and 24 h) and the length of germ tube was recorded at 12 h for *A. niger* and 24 h for *P. italicum*.

### Examination of Mycelial Growth and Micro-Morphology

To test the effect of H_2_S on mycelial growth and hyphal micro-morphology, three different sites on a Petri plate were spot-inoculated with 2 µL spore suspension (1×10^6^ spores per ml). After 3 d of water fumigation, plates were treated with NaHS solutions at MIC i.e. 0.5 mM, 2.5 mM or water (as control) for 1 d at 25°C and mycelial growth and morphological change were recorded as described by Liu et al [26].

### Detection of ROS with Fluorescence Probe in *A. niger*

A redox-sensitive fluorescent probe 2′,7′-dichlorofluorescein diacetate (DCHF-DA) was used for the analysis of intracellular ROS level according to Savi et al [27]. *A. niger* mycelia grown on PDA plates were incubated with 10 µM DCHF-DA at 37°C for 20 min in the dark. After washing the fungi with double-distilled water for three times, the fluorescence of dichlorofluorescein DCF (the oxidation product of DCHF-DA; excitation at 485 nm, emission at 530 nm) were observed using a Nikon Eclipse 80i fluorescence microscope (Nikon, Japan). Non-stained *A. niger* was used as negative control.

### Determination of SOD and CAT Activity in *A. niger*

*A. niger* mycelium grown on PDA media were suspended in 50 mM ice-cold phosphate buffer (pH 7.8, 1 mM EDTA and 1 mM PMSF) and an equal volume acid-washed glass beads (0.4–0.6 mm diameter, Sigma) were added to 1 mL of *A. niger* suspension in an Eppendorf tube. The cells were broken in a FastPrep-24 Tissue Homogenizer (MP Biomedicals, USA) for 40 s at 5500 rpm, repeated four times with 2 min on ice between intervals. The mixture was centrifuged at 12,000 rpm at 4°C for 10 min to obtain the supernatant. Activities of SOD and CAT were determined by procedures described by Giannopolitis and Ries [28] and Beers and Sizer [29] respectively. One SOD unit was defined as the amount of enzyme that inhibits the rate of nitroblue tetrazolium (NBT) reduction by 50% and one unit of CAT was defined as a decrease of 0.1 OD at 240 nm. Activity was expressed as U·mg^−1^ protein. The protein content was measured according to Bradford [30] using bovine serum albumin as standard.

### Reverse Transcriptase-Polymerase Chain Reaction (RT-PCR)

*A. niger* mycelium grown on PDA medium and an equal weight of acid-washed glass beads were added in RNAiso plus (TaKaRa, Japan) solution. The cells were bead-beaten (5500 rpm) for four rounds of 40 s with 2 min on ice between intervals. Total RNA and cDNA were obtained according to the manufacturer's instructions of RNAiso plus (TaKaRa, Japan) and PrimeScript RT Master Mix (TaKaRa, Japan), respectively. PCR conditions were as follows: initial denaturation at 94°C for 5 min, followed by cycles of 94°C for 30 s, 50°C for 30 s, and 72°C for 30 s. Actin-encoding gene (ANI_1_106134) expression was used as a control for SOD-encoding genes (ANI_1_840184, ANI_1_470064 and ANI_1_1170064) and CAT-encoding gene (ANI_1_2390104) [31]. Primers used for RT-PCR and corresponding PCR product sizes are shown as following: ANI_1_106134-For/Rev, 5′-AAAATGCTGCTGCGAATG-3′/5′-GCGGATAGCTGAACGAGAT-3′, 238 bp; ANI_1_840184-For/Rev, 5′-CGCTGCTCTTGCTGCTAT-3′/5′-GTCCTGGTTCTGTGAAATCG-3′, 153 bp; ANI_1_470064-For/Rev, 5′-CACGGATAACAGCGACTAG-3′/5′-TGAGCAGATTTGAGCACCTT-3′, 269 bp; ANI_1_1170064-For/Rev, 5′-GCTAATGCTGGACGGAACT-3′/5′-TGGTCTGTGAAGGGAAAGG-3′, 164 bp; ANI_1_2390104-For/Rev, 5′-GAATCGGCATCAACCTCC-3′/5′-CACGCTCATCCATCACTT-3′, 285 bp. The band densities of RT-PCR results were quantified with Image J software (NIH, USA).

### Antimicrobial Activity of H_2_S on Baker's Yeast, *C. albicans*, *R. oryzae*, and some Food-Borne Bacteria

*S. cerevisiae*, *C. albicans, S. aureus*, *S. typhimurium*, *L. monocytogenes*, *B. subtilis*, *B. thuringiensis*, *E. coli* and *E. aerogenes* suspensions were harvested from medium cultured for 24 h, and diluted to about 2×10^3^ cells per mL. Suspensions (100 µL) were evenly spread on YPD or nutrient agar in a Petri dish and each plate was placed in a sealed container and fumigated with H_2_S released from NaHS solutions (150 mL) at 25°C. Colony formation (CFU: Colony-Forming Units) of three replicates was recorded daily for 4 d. The lowest NaHS concentration that resulted in failure to form colonies after 2 d incubation was regarded as the minimal inhibitory concentration (MIC). For *R. oryzae*, a 2 µL spore suspension (1×10^6^ spores per mL) harvested from a 7-d culture on plate was inoculated 4 sites on a 9-cm diameter Petri dish and NaHS solutions at different concentrations (0.00, 0.05, 0.10, 0.50, 3.00 and 10.00 mM) were added in the bottom of sealed containers to fumigate *R. oryzae* at 25°C and the NaHS solutions were renewed daily. To investigate whether H_2_S treatment could kill these strains or just inhibit their growth, strains were first treated with 2.5 mM NaHS solution for 1 d and then recovered in a water atmosphere for another 6 d at 25°C.

### Statistical Analysis

Each experiment was repeated three times. Statistical significance was tested by one-way analysis of variance (ANOVA) using IBM SPSS Statistics (SPSS version 20.0), and the results were expressed as the means ± standard deviation (SD). Least significant difference test (LSD) was performed on all data following ANOVA tests to test for significant (P<0.05 or P<0.01) differences between treatments.

## Results

### Effect of H_2_S on Postharvest Condition of Fruits Inoculated with *A. niger* and *P. italicum*

**Figure 1:**
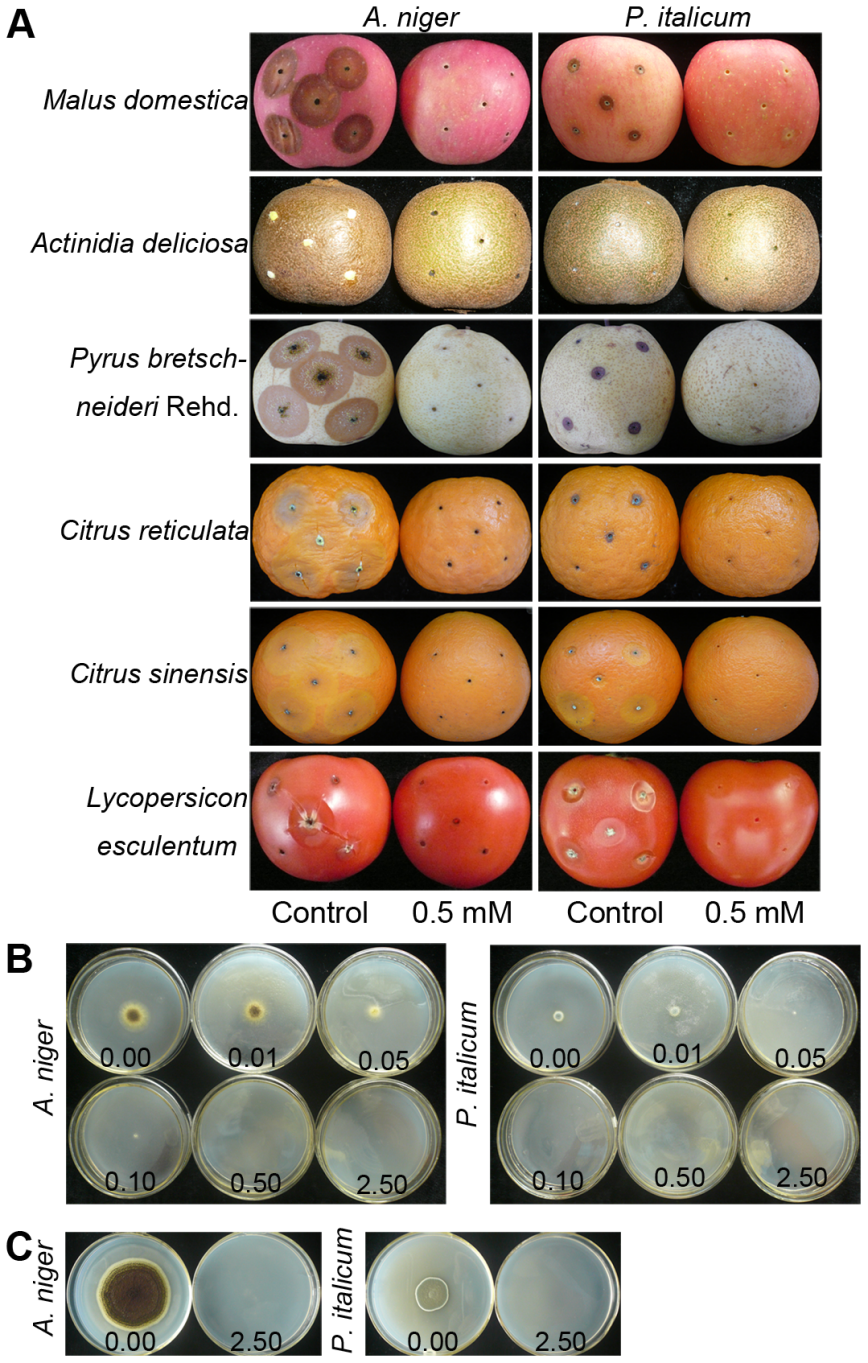
Effect of H_2_S on *A. niger* and *P. italicum* growth in inoculated fruits and on defined media. A, photographs of six kinds of postharvest fruits inoculated with *A. niger* and *P. italicum* when fumigated with 0.0 mM (control) or 0.5 mM NaHS solution for 9 d at 25°C; B, photographs of *A. niger* and *P. italicum* grown on Petri plates on defined media after fumigation with 0.00, 0.01, 0.05, 0.10, 0.50 and 2.50 mM NaHS solutions for 2 d at 25°C; C, photographs of *A. niger* and *P. italicum* after 1 d of 2.5 mM NaHS treatment and recovered in water fumigation for another 8 d at 25°C.

As shown in Figure 1A, H_2_S released by 0.5 mM NaHS solution effectively controlled fruit decay caused by *A. niger* and *P. italicum*. After infection with *A. niger* or *P. italicum*, apples, pears and tomatoes from control condition began to decay at day 3, and kiwifruits, sweet oranges and mandarins at day 5, while fruits from H_2_S treatment remained decay-free (data not shown). When stored for 9 d, obvious signs of decay were found in control fruits, whereas lesion diameters of fruits from H_2_S treatment remained basically unchanged (Figure 1A).

### Effect of H_2_S on Growth of Cultured *A. niger* and *P. italicum*

As shown in Figure 1B and Table 1, H_2_S significantly (P<0.05) inhibited colony growth of *A. niger* or *P. italicum* when grown on YPD plates in a dose-dependent manner. For *A. niger* at day 2, colony diameter with 0.1 mM NaHS was approximately 16% of that in control. In contrast, colony formation of *P. italicum* was not observed when NaHS solution was increased to 0.1 mM at day 2 (Figure 1B and Table 1). The data in Table 1 show that MIC, the minimum concentration of NaHS that resulted in failure of colony formation after 4 d of incubation, for both *A. niger* or *P. italicum* was 0.5 mM. In addition, when *A. niger* or *P. italicum* were treated with 2.5 mM NaHS for 1 day and recovered in a water atmosphere for another 8 d, growth of both fungi was completely suppressed (Figure 1C).

**Table 1:** Colony diameter of *A. niger* and *P. italicum* after exposure to H_2_S at 25°C.

Strains	Treatment (mM NaHS)	Treatment time			
		2 d	4 d	6 d	8 d
		Diameter (mm)	Diameter (mm)	Diameter (mm)	Diameter (mm)
*A. niger*	0.00	27.1±0.9 a	41.5±3.1 a	51.4±2.8 a	58.6±2.8 a
	0.01	24.2±1.0 b	39.6±1.7 b	47.0±0.7 b	54.7±0.7 b
	0.05	17.8±0.3 c	39.5±1.5 b	48.3±0.5 b	54.9±2.0 b
	0.10	4.2±0.5 d	32.8±1.0 c	43.3±1.5 c	52.9±1.3 b
	0.50	0 e	0 d	0 d	0 c
	2.50	0 e	0 d	0 d	0 c
*P. italicum*	0.00	9.2±0.3 a	17.3±1.1 a	22.2±1.3 a	25.3±1.3 a
	0.01	8.5±0.7 b	16.7±0.5 a	21.2±1.0 a	24.0±1.4 a
	0.05	3.8±0.5 c	12.4±0.1 b	18.3±0.7 b	21.0±1.0 b
	0.10	0 d	10.0±0.2 c	16.5±0.6 c	20.4±1.3 b
	0.50	0 d	0 d	0 d	0 c
	2.50	0 d	0 d	0 d	0 c

Different letters mean significance of difference between the treatments (P<0.05, ANOVA, LSD). Diameter: colony diameter.

### Effect of H_2_S on Spore Germination and Germ Tube Elongation

The data in Table 2 show that exposure to H_2_S significantly (P<0.05) inhibited spore germination and germ tube elongation in *A. niger* and *P. italicum* in a dose-dependent manner. At 12 h, germination percentages of *A. niger* spores in 0.01 mM and 0.05 mM NaHS were approximately 86% and 4.5% of control, respectively, while no germination of *P. italicum* spores occurred in 0.1 mM NaHS. At 24 h, more spores of both fungi germinated at H_2_S concentrations below 0.1 mM NaHS. However, 0.5 mM and 2.5 mM NaHS totally inhibited spore germination in *A. niger* and *P. italicum*. The length of germ tubes decreased by 93% at 12 h for both *A. niger* and *P. italicum* at 24 h in 0.05 mM NaHS treatment compared with that of water control. Complete inhibition of spore germination and germ tube elongation occurred in the presence of NaHS concentrations higher than 0.5 mM.

**Table 2:** Spore germination and germ tube elongation of *A. niger* and *P. italicum* after exposure to H_2_S at 25°C.

Strains	Treatment (mM NaHS)	Treatment time				
		6 h	12 h		24 h	
		Germ (%)	Germ (%)	El (µm)	Germ (%)	El (µm)
*A. niger*	0.00	0 a	73.3±1.8 a	37.3±5.0 a	100±0.0 a	–
	0.01	0 a	62.9±2.9 b	12.6±1.5 b	100±0.0 a	–
	0.05	0 a	3.3±0.5 c	2.7±0.9 c	100±0.0 a	–
	0.10	0 a	0 d	0 c	77.8±2.5 b	–
	0.50	0 a	0 d	0 c	0 c	–
	2.50	0 a	0 d	0 c	0 c	–
*P. italicum*	0.00	0 a	53.0±2.1 a	–	100±0.0 a	155.6±14.1 a
	0.01	0 a	0 b	–	100±0.0 a	116.4±12.5 b
	0.05	0 a	0 b	–	84.5±2.2 b	10.7±2.1 c
	0.10	0 a	0 b	–	10.1±2.6 c	4.5±0.7 cd
	0.50	0 a	0 b	–	0 d	0 d
	2.50	0 a	0 b	–	0 d	0 d

Different letters mean significance of difference between the treatments (P < 0.05, ANOVA, LSD). Germ: spore germination percentage; El: germ tube elongation. The symbol “–” stands for not determined at this time point.

### Effect of H_2_S on Mycelial Growth and Hyphal Morphology

**Figure 2:**
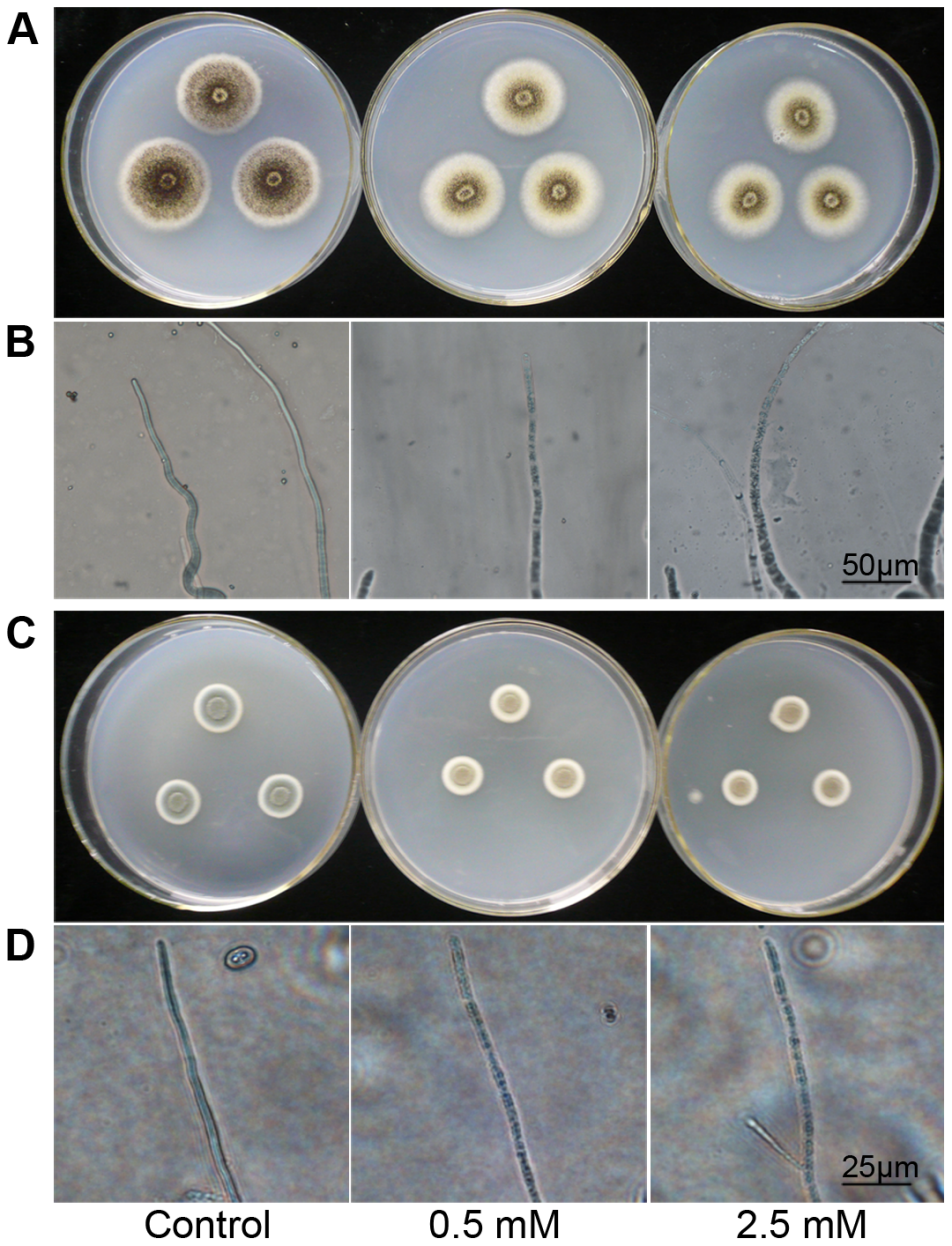
Effect of H_2_S fumigation on mycelial growth and micro-morphology of *A. niger* and *P. italicum* grown on defined media. Colonies were cultured for 3°C, and then exposed to H_2_S released from 0.5 mM and 2.5 mM NaHS for 1 d. A and B, mycelial growth and micro-morphology of *A. niger*; C and D, mycelial growth and micro-morphology of *P. italicum*.

Mycelial growth and hyphal morphology of *A. niger* and *P. italicum* are presented in Figure 2. As shown in Figure 2A and C, H_2_S treatment reduced mycelial diameter compared with control. To further test the effect of H_2_S on mycelial micro-morphology, mycelia were examined by light microscopy and morphological deformities (Figure 2B and D) were found. Abnormal contraction of mycelial cytoplasm appeared in *A. niger* and *P. italicum* when fumigated with 0.5 mM or 2.5 mM NaHS for as short as 1 d. For *A. niger*, 2.5 mM treatment even led to the fragmentation of mycelial cytoplasm (Figure 2B).

### Effect of H_2_S on ROS Level in *A. niger*

**Figure 3:**
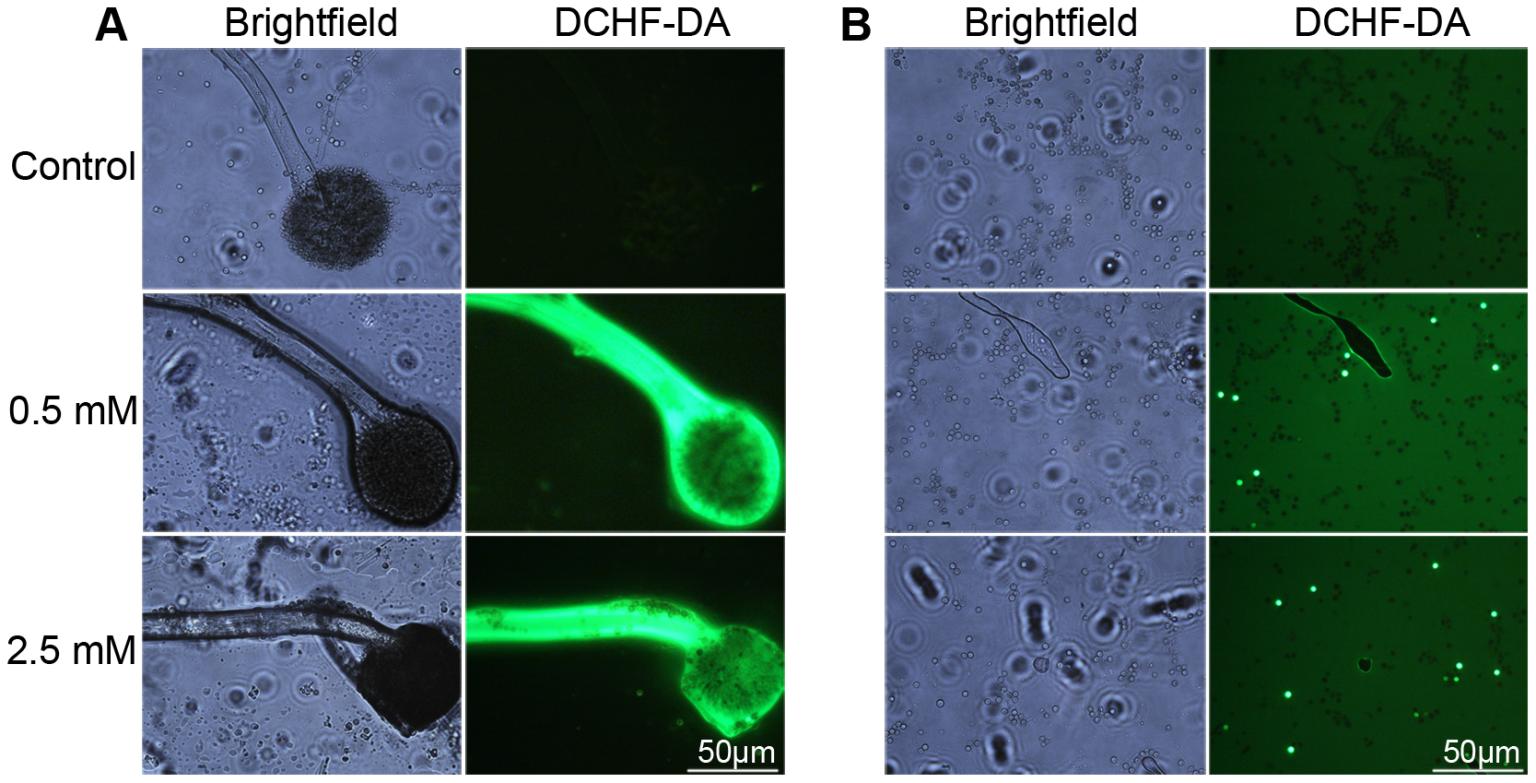
Effect of H_2_S fumigation on ROS level in *A. niger*. *A. niger* cells in Figure 2 were used for ROS detection. A, ROS staining in sporangia and sporangiophores; B, ROS staining in *A. niger* spores. Left parts of A and B shows the bright field images, and right parts fluorescence images.

The oxidant sensitive probe 2′,7′-dichlorofluorescein diacetate (DCHF-DA) was used to assess the ROS level in *A. niger*. Treatment with 0.5 mM or 2.5 mM NaHS solution significantly increased ROS level in the sporangia and sporangiophores (Figure 3A) compared with water control. However, not all spores from H_2_S treatments showed a higher level of ROS (Figure 3B), which might be due to the fact that only peripheral spores in sporangia were exposed to H_2_S.

### Effect of H_2_S on Enzyme Activities and Expression of SOD and CAT Genes in *A. niger*

**Figure 4:**
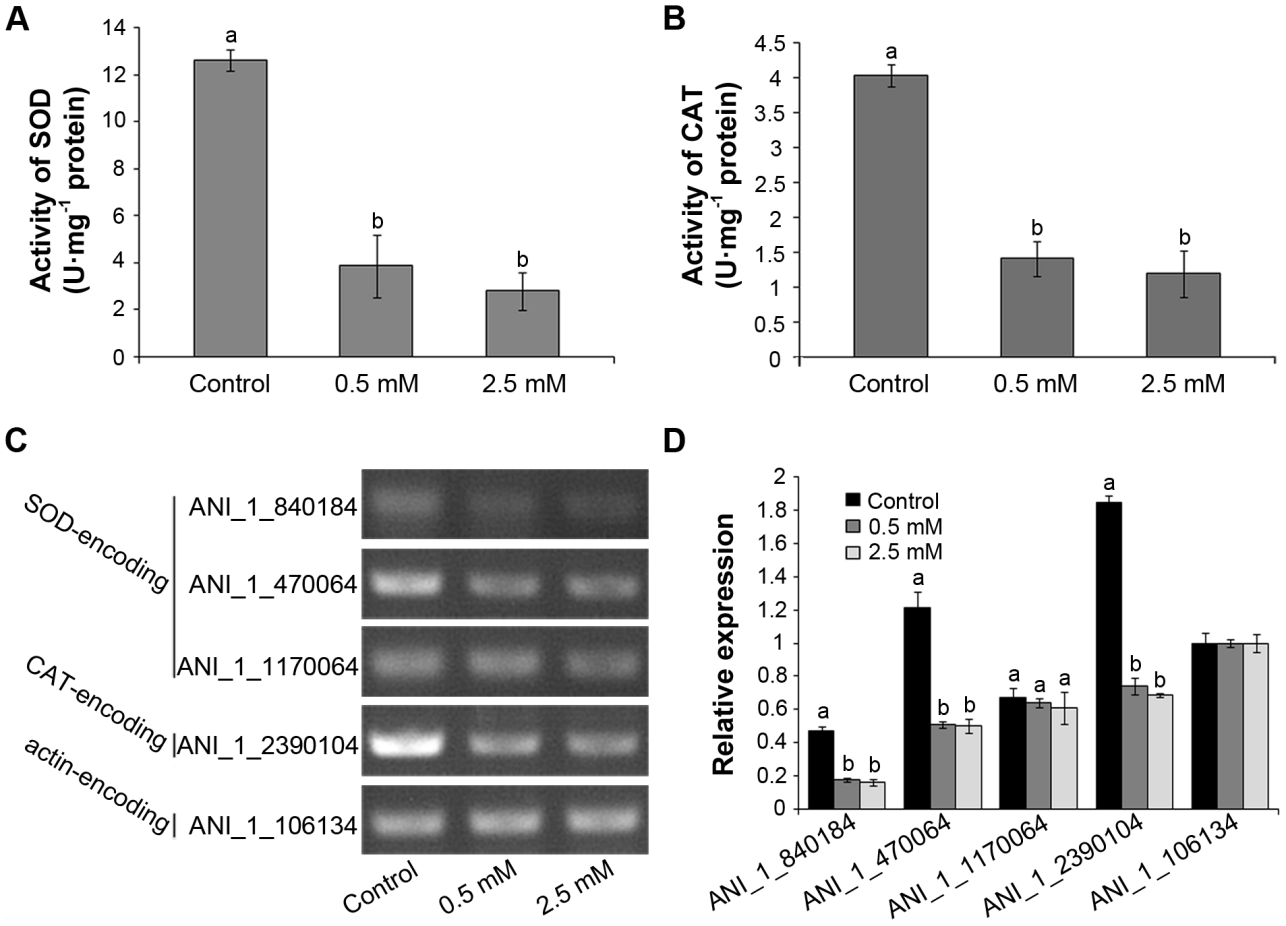
Effect of H_2_S fumigation on enzyme activities and gene expression of SOD and CAT in *A. niger*. Colonies were cultured for 3°C, and then exposed to H_2_S released from 0.5 mM and 2.5 mM NaHS for 1 d. A, SOD activity in *A. niger*; B, CAT activity in *A. niger*; C, RT-PCR results to analyze gene expressions of SOD and CAT in *A. niger*. D, relative expression levels by quantifying RT-PCR results in C (with the actin-encoding gene value [ANI_1_106134] set as 1.0) with Image J software. Different letters mean significance of difference between the treatments (P<0.01, ANOVA, LSD).

As shown in Figure 4A and B, H_2_S treatments significantly (P<0.01) decreased both SOD and CAT activities in *A. niger* compared with the control. SOD activity in H_2_S treatment was reduced by 69% (0.5 mM NaHS) and 78% (2.5 mM NaHS) compared with control, respectively. Similarly, CAT activity in H_2_S treatments was only 35% (0.5 mM NaHS) and 30% (2.5 mM NaHS) of that in control, respectively. The expression of SOD and CAT genes was examined by RT-PCR and the data shown in Figure 4C and D. Compared with controls, exposure of *A. niger* to H_2_S significantly decreased the expression of the SOD-encoding genes ANI_1_840184 and ANI_1_470064, and the CAT-encoding gene ANI_1_2390104, while no obvious change appeared in the transcript of the SOD-encoding gene ANI_1_1170064. The enzyme activity and gene expression assays of SOD and CAT indicated that H_2_S negatively regulated antioxidant system in *A. niger*.

### Effect of H_2_S on Baker's Yeast, *C. albicans*, *R. oryzae* and some Food-Borne Bacteria

**Figure 5:**
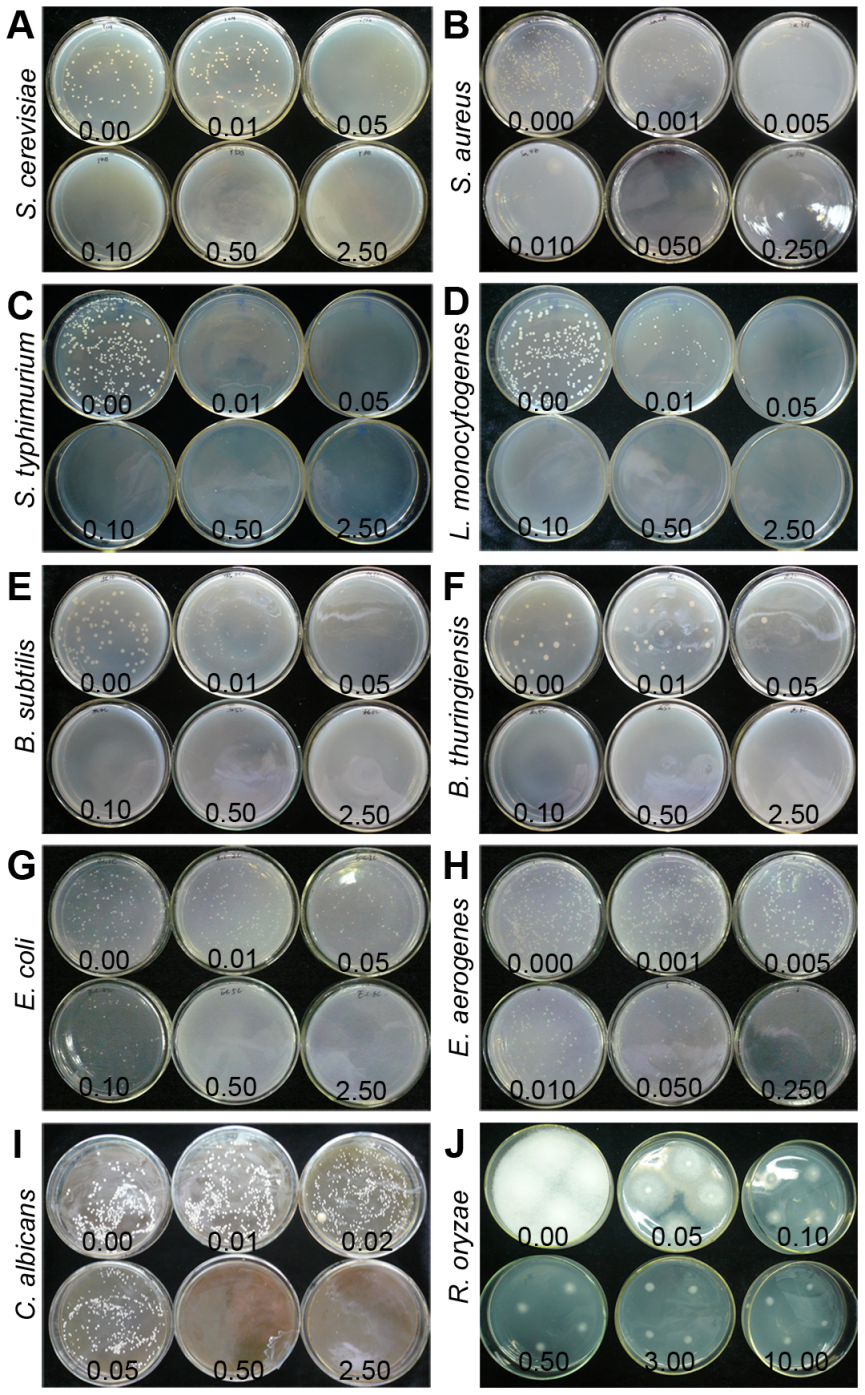
Effect of H_2_S fumigation on grown of baker's yeast, *C. albicans*, *R. oryzae*, and some food-borne bacteria growth on defined media. A–F, photographs of *S. cerevisiae*, *S. aureus*, *S. typhimurium*, *L. monocytogenes*, *B. subtilis* and *B. thuringiensis* after 3 d exposure to H_2_S released from different concentrations of NaHS at 25°C; G–H, photographs of *E. coli* and *E. aerogenes* after exposure to H_2_S for 1 d; I–J, photographs of *C. albicans* and *R. oryzae* after exposure to H_2_S for 4 d and 7 d, respectively.

**Figure 6:**
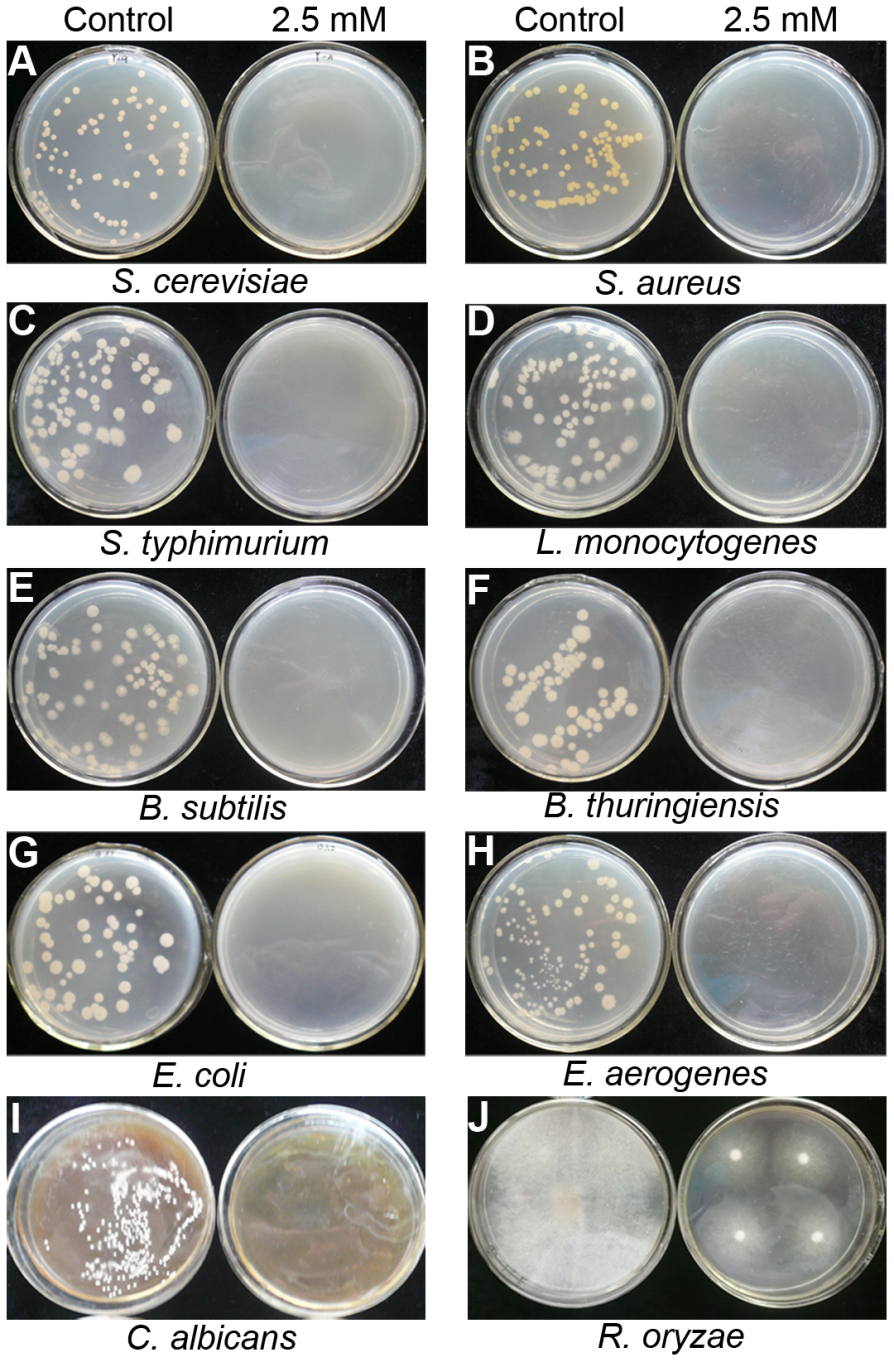
Effect of short term H_2_S exposure to baker's yeast, *C. albicans*, *R. oryzae*, and some food-borne bacteria grown on defined media. After 1°C, the photographs were taken. A–H show photographs of *S. cerevisiae*, *S. aureus*, *S. typhimurium*, *L. monocytogenes*, *B. subtilis*, *B. thuringiensis*, *E. coli* and *E. aerogenes*, respectively; I–J show photographs of *C. albicans* and *R. oryzae* recovered in water atmosphere for 2 and 4 d after 2.5 mM NaHS treatment for 1 d.

To study possible effect of H_2_S on baker's yeast, *C. albicans*, *R. oryzae* and some food-borne bacteria, we examined the growth of *S. cerevisiae*, *C. albicans*, *R. oryzae*, *S. aureus*, *S. typhimurium*, *L. monocytogenes*, *B. subtilis*, *B. thuringiensis*, *E. coli* and *E. aerogenes* grown on culture media. The data in Figure 5 and Table 3 show that H_2_S had a significant (P<0.05) antimicrobial effect on *S. cerevisiae*, *C. albicans* and on bacteria in a dose-dependent manner. *S. aureus* was the most sensitive to H_2_S treatment. The MIC was determined to be 0.01 mM for *S. aureus*, 0.05 mM for *S. cerevisiae*, *S. typhimurium*, *L. monocytogenes* and *B. subtilis*, 0.1 mM for *B. thuringiensis*, 0.25 mM for *E. aerogenes*, 0.5 mM for *C. albicans* and 2.5 mM for *E. coli*. In contrast, *R. oryzae* showed high resistance to H_2_S fumigation though H_2_S did inhibit the colony growth of *R. oryzae*, and the MIC might be very high (Figure 5). Moreover, after 1 d exposure to 2.5 mM NaHS, no colony formation was observed in any strains after recovery in a water atmosphere for a further 6 d (Figure 6) except *R. oryzae*, suggesting that high concentrations of H_2_S exhibited a microbicidal role rather than just inhibiting the growth of microbes.

**Table 3:** Colony formation of yeasts and bacteria after exposure to H_2_S at 25°C.

Strains	Treatment (mM NaHS)	Treatment time			
		1 d	2 d	3 d	4 d
		Number (CFU)	Number (CFU)	Number (CFU)	Number (CFU)
					
*S. cerevisiae*	0.00	0 a	81.0±7.2 a	81.0±7.2 a	81.0±7.2 a
	0.01	0 a	85.7±9.0 a	85.7±9.0 a	85.7±9.0 a
	0.05	0 a	0 b	20.5±2.1 b	47.5±4.9 b
	0.10	0 a	0 b	0 c	0 c
	0.50	0 a	0 b	0 c	0 c
	2.50	0 a	0 b	0 c	0 c
					
*S. aureus*	0.000	0 a	73.0±2.8 a	351.5±3.5 a	351.5±3.5 a
	0.001	0 a	20.0±1.4 b	114.0±2.8 b	114.0±.8 b
	0.005	0 a	0.5±0.7 c	18.5±0.7 c	30.0±1.4 c
	0.010	0 a	0 c	11.5±0.7 d	20.5±2.1 d
	0.050	0 a	0 c	0 e	0 e
	0.250	0 a	0 c	0 e	0 e
*S. typhimurium*	0.00	357.0±24.0 a	368.5±19.1 a	368.5±19.1 a	368.5±19.1 a
	0.01	0 b	8.0±2.8 b	26.5±2.1 b	42.5±0.7 b
	0.05	0 b	0 b	0.5±0.7 c	3±4.2 c
	0.10	0 b	0 b	0 c	0 c
	0.50	0 b	0 b	0 c	0 c
	2.50	0 b	0 b	0 c	0 c
*L. monocytogenes*	0.00	0 a	316.5±20.5 a	316.5±20.5 a	316.5±20.5 a
	0.01	0 a	40.0±9.9 b	62.5±14.8 b	72.5±12.0 b
	0.05	0 a	0 c	2.0±1.4 c	2.5±2.1 c
	0.10	0 a	0 c	0 c	0 c
	0.50	0 a	0 c	0 c	0 c
	2.50	0 a	0 c	0 c	0 c
*B. subtilis*	0.00	0 a	78.3±8.7 a	78.3±8.7 a	78.3±8.7 a
	0.01	0 a	47.0±18.3 b	48.7±18.0 b	56.3±9.7 b
	0.05	0 a	0 c	0.7±0.6 c	0.7±0.6 c
	0.10	0 a	0 c	0 c	0 c
	0.50	0 a	0 c	0 c	0 c
	2.50	0 a	0 c	0 c	0 c
*B. thuringiensis*	0.00	8.3±3.5 a	21.0±2.0 a	21.7±1.2 a	23.0±2.0 a
	0.01	6.0±1.0 a	16.7±4.2 b	19.3±3.1 a	21.0±1.0 b
	0.05	0.7±0.6 b	0.7±0.6 c	0.7±0.6 b	0.7±0.6 c
	0.10	0 b	0 c	0 b	0 c
	0.50	0 b	0 c	0 b	0 c
	2.50	0 b	0 c	0 b	0 c
*E. coli*	0.00	143.7±11.6 a	193.0±24.8 a	193.0±24.8 a	193.0±24.8 a
	0.01	102.7±16.0 b	157.0±14.7 b	157.0±14.7 b	157.0±14.7 b
	0.05	61.3±0.6 c	128.3±8.3 c	128.3±8.3 c	128.3±8.3 c
	0.10	38.0±8.5 d	94.0±11.3 d	127.7±8.1 c	127.7±8.1 c
	0.50	0 e	43.0±23.9 e	83.0±22.9 d	97.7±10.1 d
	2.50	0 e	0 f	0 e	0 e
*E. aerogenes*	0.000	225.5±4.9 a	236.5±4.9 a	236.5±4.9 a	236.5±4.9 a
	0.001	205.5±7.8 b	218.0±7.1 a	218.0±7.1 b	218.0±7.1 b
	0.005	114.0±4.2 c	141.5±2.1 b	141.5±2.1 c	141.5±2.1 c
	0.010	71.0±1.4 d	137.5±3.5 b	137.5±3.5 c	137.5±3.5 c
	0.050	37.5±2.1 e	89.0±1.4 c	120.0±2.8 d	120.0±2.8 d
	0.250	0 f	0 d	0 e	0 e
*C. albicans*	0.00	–	–	–	401.0±14.8 a
	0.01	–	–	–	378.7±10.2 a
	0.02	–	–	–	325.3±8.3 b
	0.05	–	–	–	135.6±11.5 c
	0.50	–	–	–	0 d
	2.50	–	–	–	0 d

Different letters mean significance of difference between the treatments (P<0.05, ANOVA, LSD). CFU: Colony-Forming Units. The symbol “–” stands for not determined at this time point.

## Discussion

The results reported in this work show the possibility of an alternative strategy for postharvest fruit storage, based on exposure of infected fruit to H_2_S released by NaHS. Our previous work found that exogenous H_2_S could extend the postharvest life of strawberry, fresh-cut kiwifruit, broccoli and pear [15], [20], [21], [24], while there are fewer data on an antifungal role of H_2_S against postharvest pathogens. Haneklaus et al. [32] reported that uptake of 10 µM/h H_2_S by the pathogen would produce a fungicidal effect. It has also been shown that endogenous H_2_S release showed a significant rise when agricultural crops suffered from fungal infection [33], [34], suggesting an important role of H_2_S in plant defense against fungal attack. In this study, we found an effective antifungal effect of H_2_S to the postharvest pathogens *A. niger* and *P. italicum* inoculated on fruits and grown on defined medium (Figure 1 and Table 1). Consistent with these observations, we previously reported that H_2_S plays as a role as a fungicide to the pear pathogens *P. expansum* and *A. niger* and prolongs postharvest storage of fresh-cut pears [24].

We also studied the toxicity of H_2_S to baker's yeast, the human pathogen *C. albicans* and several bacteria, and find that H_2_S released by 2.5 mM NaHS plays a microbicidal role rather than just inhibiting the growth of molds, yeasts and bacteria (Figure 1C, Figure 6 and Table 3), while *R. oryzae* is more resistant to H_2_S. Our work also shows that H_2_S exerts its antifungal effect by affecting various aspects of fungal growth, including: 1) inhibiting spore germination and germ tube elongation (Table 2), 2) retarding mycelial growth (Figure 2A and C), and 3) causing abnormal contraction even fragmentation of mycelial cytoplasm (Figure 2B and D). Similar to the effect of H_2_S towards mycelial cytoplasm that we observed, Senthilkumar et al. [35] also observed such deformities in the mycelia of *Paenibacillus* sp. HKA-15 when exposed to antibiotics.

Recently, Lai et al. [19] reported that NO could inhibit the germination of *P. expansum* spores and induce an increase of intracellular ROS which might result from increased ROS formation and decreased ROS detoxification. In the present study, we found that H_2_S could increase intracellular ROS in sporangia and sporangiophores as well as in spores of *A. niger* (Figure 3A and B). It is unclear that whether H_2_S directly induces the formation of ROS in *A. niger*. However, Eghbal et al. [36] have reported that H_2_S exerts its cytotoxic effect against hepatocytes via inducing ROS formation and the overall reaction could be written as: *n*S^2−^ + 2*n*O_2_ → Sn + 2*n*O_2_^−^. In addition, H_2_S has also been found to induce oxidative damage in *Glycera dibranchiate* and mammalian cells [37], [38]. Therefore, we speculate that H_2_S might directly induce ROS generation in *A. niger* and excessive ROS subsequently causes oxidative damage to molecules crucial for mycelial growth and spore germination. Antioxidant enzymes such as SOD and CAT also play important roles in ROS elimination in response to oxidative stress [39]. Our results also show that H_2_S could inhibit gene expression (Figure 4C and D) and decrease enzyme activities of SOD and CAT (Figure 4A and B), which might contribute to the increased ROS level observed in H_2_S-treated *A. niger*. In contrast, the studies in postharvest storage of fruits and vegetables reveal that H_2_S can eliminate ROS accumulation by improving the endogenous antioxidant system [15], [20], [21], [22], [24]. Thus H_2_S exhibits different effects on microbes and plants which might be due to different tolerance to H_2_S. Besides, the phenomenon that endogenous H_2_S released when agricultural crops suffered from fungal infection [33], [34] also supports our hypothesis. Endogenous H_2_S in Gram-negative and Gram-positive bacteria is also required for antibiotic tolerance by elevating their antioxidant capacity [40]. In our study, we find that exogenous H_2_S application effectively inhibits the growth of a series of bacteria (Figure 5), which can be attributed to the relatively higher level of applied H_2_S than lower level in endogenous production.

The evaluation of postharvest fungi *A. niger*, *P. italicum* and *R. oryzae*, yeasts and several food-borne bacteria strongly suggests the possible commercial value of H_2_S fumigation to reduce postharvest spoilage and food storage. In this work, we also show that a fungicidal role of H_2_S might be associated with increased ROS accumulation in H_2_S-treated fungi. Further studies involving oxidative damage will help to better understand the ROS-related mechanism by which H_2_S inhibits the growth of postharvest pathogens.
